# The mediating role of emotional intelligence in the relationship between sexual health literacy and sexual communication self-efficacy among married nursing students

**DOI:** 10.1186/s12912-025-03157-y

**Published:** 2025-06-17

**Authors:** Shaimaa Mohamed Amin, Heba Emad El-Gazar, Mohamed Ali Zoromba, Nagwa Ibrahim ELfeshawy, Toha Ali ELsayed Abo-Hatab, Hanan Hosni El-Sherbini, Mohamed Hussein Ramadan Atta

**Affiliations:** 1 Lecturer of Community Health Nursing, Faculty of Nursing, Damanhour University, Damanhour, Egypt; 2 Nursing Administration Department, Faculty of Nursing, Port Said University, Port Said, Egypt; 3 College of Nursing, Prince Sattam Bin Abdulaziz University, Al-Kharj, Saudi Arabia; 4 Psychiatric and Mental Health Nursing Department, Faculty of Nursing, Mansoura University, Mansoura, Egypt; 5 Women’s Health and Midwifery Nursing, Faculty of Nursing, Mansoura University, Mansoura, Egypt; 6 Maternal and Pediatric Nursing, College of Nursing, King Khalid University, KSA, Saudi Arabia; 7 Lecturer of Maternal and Neonatal Health Nursing, Faculty of Nursing, Tanta University, Tanta, Egypt; 8 Community Health Nursing, Faculty of Nursing, Alexandria University, Alexandria City, Egypt; 9 Nursing Department, College of Applied Medical Sciences, Prince Sattam Bin Abdulaziz University, Wadi Addawasir, Saudi Arabia; 10 Lecturer of Psychiatric and Mental Health Nursing, Psychiatric and Mental- Health Nursing Department, Faculty of Nursing, Alexandria University, Alexandria City, Egypt

**Keywords:** Sexual health literacy, Emotional intelligence, Sexual communication self-efficacy, Nursing students

## Abstract

**Background:**

Sexual health literacy is essential for improving communication about sexual health, especially among married nursing students. Emotional intelligence may play a significant role in facilitating this communication by mediating the relationship between sexual health literacy and sexual communication self-efficacy.

**Objective:**

This study aimed to examine the relationships between sexual health literacy, emotional intelligence, and sexual communication self-efficacy among married nursing students and assess the mediating role of emotional intelligence.

**Methods:**

A cross-sectional study was conducted with 340 married female nursing students at the Faculty of Nursing, Tanta University, Egypt. Data were collected using validated scales for sexual health literacy, emotional intelligence, and sexual communication self-efficacy. Statistical analyses were performed, including t-tests, ANOVA, Pearson correlation, and mediation analysis via the PROCESS macro (Model 4). Bias-corrected bootstrapping with 5,000 resamples was applied to test for the mediating effects of emotional intelligence.

**Results:**

The results revealed no significant differences in sexual health literacy, sexual communication self-efficacy, or emotional intelligence across demographics. Pearson correlations demonstrated that sexual health literacy was positively correlated with sexual communication self-efficacy (*r* = .388, *p* < .01) and emotional intelligence (*r* = .560, *p* < .01). Mediation analysis showed that emotional intelligence significantly mediated the relationship between sexual health literacy and sexual communication self-efficacy (B = 0.04,). The effect of sexual health literacy on sexual communication self-efficacy was significant (B = 0.19, *p* < .001), with emotional intelligence accounting for a portion of this relationship.

**Conclusion:**

This study highlights the importance of sexual health literacy in promoting effective communication about sexual health. Emotional intelligence plays a key mediating role, suggesting that enhancing both sexual health literacy and emotional intelligence could improve sexual communication self-efficacy among married nursing students. These findings suggest potential interventions to integrate emotional intelligence training into sexual health education programs to enhance communication outcomes.

**Clinical trial number:**

Not applicable.

## Introduction

In adulthood, academic students often face significant life transitions, including the possibility of early marriage and parenthood [1]. During this critical period, they must have a strong foundation in reproductive knowledge. This knowledge not only helps them understand their sexual health but also equips them to navigate the responsibilities of parenthood [2]. Early marriage can present challenges for new parents, making it important to emphasize the role of sexual education in fostering open communication between partners. By promoting healthy discussions about intimacy, reproductive health, and family planning, young adults can build stronger, more supportive relationships, crucial for emotional well-being and successful family dynamics [3].

UNESCO, 2023 reported that only 70% of youth in sub-Saharan Africa lack crucial knowledge as they approach puberty and start menstruation. The issues of early marriage and unintended pregnancy pose significant global challenges to girls’ health and education, with pregnancy rates in East and Southern Africa ranging between 15 and 25%, among the highest worldwide. These factors highlight the importance of delivering quality, comprehensive sex education to enhance learners’ health, knowledge, and empowerment [4].

Sexual health literacy (SHL) encompasses a range of knowledge, beliefs, attitudes, motivations, and personal abilities in accessing, interpreting, evaluating, and using sexual health information in daily life. This literacy empowers individuals to make informed decisions and effect changes in their sexual lives [5]. According to the World Health Organization, SHL plays a crucial role in achieving several Sustainable Development Goals, including the eradication of poverty and hunger, the promotion of equal education, gender equality, the empowerment of women and girls, economic growth, and reducing inequality [6, 7]. Promoting SHL, especially among women, serves as an effective strategy for mitigating the burden of sexual health-related diseases. By equipping women with knowledge about sexual health risks and promoting their agency in sexual well-being, SHL can reduce unintended pregnancies and sexually transmitted infections while fostering healthier sexual relationships [7, 8].

A systematic review recommended a pressing need for services that promote sexual health literacy, which should be factored into policymaking, planning, and designing health programs [9]. In addition, Nematzadeh et al. 2024 surveyed sample included both male and female students, indicating 20.5% of the participants demonstrated limited sexual health literacy. In more depth, engagement with social media for sexual health information, the source of information from various channels and online platforms, and educational level were linked to increased sexual health literacy [10].

Sexual communication is often emphasized as a crucial factor that affects sexual health behaviors. Sexual behaviors can be analyzed from multiple perspectives, including individual factors (e.g., emotion dysregulation, impulsivity), relational factors (e.g., attachment, communication), societal factors (e.g., norms, gender roles), and situational factors (e.g., alcohol use, dating apps). Proximal factors such as sexual communication, self-efficacy, confidence in discussing sexual topics with a partner, and risk norms are significant because they represent key targets for interventions. Distal factors like personality and attachment styles help explain the underlying causes of proximal behaviors, informing theoretical frameworks and aiding early risk assessment in prevention programs. However, the interplay between proximal and distal factors remains underexplored [11]. Self-efficacy, which refers to an individual’s belief in their capacity to succeed in specific behaviors [12], is a key predictor of sexual risk-taking. Prior studies indicate that higher self-efficacy correlates with better sexual health outcomes [13]. Nonetheless, few studies have focused on sexual communication self-efficacy, particularly among young adults, despite its critical role in ensuring safer sexual practices [14].

Emotional intelligence (EI) generally refers to adaptive skills in managing impulses and handling stress, as well as the ability to comprehend and manage both one’s own and others’ emotions. This includes capacities like empathy, emotional self-perception, and emotion regulation [15]. EI is conceptualized in two primary ways: an ability or a trait. Mayer and colleagues introduced the ability model, which defines EI as a cognitive skill reflecting individual differences in processing emotional information [16].

A notable correlation was found between emotional intelligence and sexual functioning in fertile individuals, with the average scores of all EI components except for interpersonal relationships, flexibility, responsibility, empathy, and self-expression being significantly linked to sexual performance [17]. While research has explored EI’s influence on sexual performance [16, 18], few studies have focused explicitly on nursing students. This population may possess unique perspectives on sexual health literacy and communication due to their healthcare background. Moreover, although studies have demonstrated correlations between EI and sexual functioning, there is a limited exploration of how EI affects sexual communication self-efficacy, the ability to discuss sexual needs with a partner confidently [17].

Additionally, gender differences in EI suggest that women may exhibit stronger empathy and social skills, but how these differences influence sexual communication among married individuals remains unclear [19]. Furthermore, while previous research emphasizes the importance of interpersonal and emotional factors in sexual issues [20], little attention has been given to how these factors play out in marital relationships, particularly among nursing students balancing professional and personal roles. Finally, the relationship between EI and sexual health literacy has not been fully explored, especially in terms of how emotional regulation impacts one’s ability to acquire and communicate sexual health knowledge. These gaps highlight the need for a study examining the intersection of emotional intelligence, sexual health literacy, and sexual communication self-efficacy among married nursing students.

By focusing on married nursing students, our research could contribute to a deeper understanding of the role of EI in enhancing marital relationships, particularly in sexual communication and mutual satisfaction. As communication is a critical factor in marital harmony, exploring the impact of EI could provide valuable implications for interventions designed to strengthen these skills among healthcare professionals, who may face unique stressors and time constraints that challenge their relationships.

Moreover, by integrating EI with sexual health literacy, this study could pave the way for more holistic approaches to addressing sexual issues, emphasizing the importance of emotional factors alongside knowledge and communication. The findings could have practical implications for developing educational interventions for nursing students, promoting professional competencies, and enhancing personal well-being and relationship satisfaction.

## Subjects and methods

### Study design

This study employed a cross-sectional survey design, following the guidelines of the STROBE checklist.

### Setting

The study was conducted at the Faculty of Nursing, Tanta University, in Gharbia Governorate, Egypt. The faculty encompasses nine specialized scientific departments that cover a wide range of nursing disciplines. The faculty’s undergraduate and graduate programs operate under a credit hours system, providing a well-structured framework for tracking academic progress and enabling thorough evaluation of educational outcomes.

### Sample size and study participants

The target group for this study comprised married female nursing students, including undergraduates, interns, and postgraduates. Participants were eligible if they were currently married, actively enrolled in an undergraduate or postgraduate nursing program at the university, and willing to participate in the study. Students were excluded if they were on academic leave, not actively enrolled in the nursing program, had previously participated in similar studies to minimize response bias, or had a documented history of severe mental health disorders that could significantly affect emotional or communicative functioning.

Additionally, students with chronic physical illnesses that significantly impact cognitive, emotional, or communication abilities were excluded to ensure that the study results were not influenced by underlying health conditions. These criteria were designed to maintain the homogeneity of the sample and enhance the validity of the study findings.

The sample size was determined using the G*Power 3.1.9.7 statistical test program [21]. The calculation parameters included a power of 0.95, an alpha of 0.05, and a moderate effect size of 0.15. Based on these parameters, the minimum required sample size was calculated to be at least 172 participants. The final sample size comprised 340 nursing students.

### Measurements of interest

#### The demographic form

The data collected included participants’ age, academic year, place of residence, employment status, family income, age at marriage, duration of marriage and the number of children. It also encompassed the spouse’s age and level of education.

### The sexual health literacy for adults (SHELA) questionnaire

It was developed by Maasoumi et al. [22] to assess sexual health literacy among adults [22]; the questionnaire utilized in the current study was validated by Panahi et al. (2022) [23]. It comprises 39 items divided into three subscales: Reading and Understanding (17 items), Evaluation and Application of Information (11 items), and Skills of Access (11 items). Examples of items include: “It is easy for me to read educational materials related to couples’ sexual relations and the factors that affect them” for the Reading and Understanding subscale; “If my spouse has a sexual problem, I will go with him for sexual counseling” for the Evaluation and Application of Information subscale; and “I can get information about communicable diseases from different sources” for the Skills of Access subscale. Each item is rated on a five-point Likert scale from strongly disagree (1) to strongly agree (5), with higher total scores reflecting greater sexual health literacy. According to Panahi et al. [23], the questionnaire demonstrated strong validity and reliability. Construct validity was supported by exploratory factor analysis, which identified three factors, and confirmatory factor analysis yielded favorable fit indices (RMSEA = 0.071, CFI = 0.928, TLI = 0.919, SRMR = 0.041, X2/pdf = 2.501) [23]. The internal consistency was excellent, with a Cronbach’s alpha of 0.981. In this study, the scale showed high reliability with a Cronbach’s alpha 0.941. Validity post-translation into Arabic was confirmed through exploratory factor analysis, which initially revealed factor loadings between 0.50 and 0.85, improving to 0.65 to 0.97 after varimax rotation. These values surpassed the 0.40 threshold, accounting for 75.230% of the total variance. The Kaiser–Meyer–Olkin (KMO) measure was substantial at 0.950, and Bartlett’s test of sphericity was highly significant (*p* ≤ .001), confirming the adequacy of the data for factor analysis. Consequently, all items on the scale were retained.

### The sexual communication self-efficacy scale (SCSES)

The scale was developed by Quinn-Nilas et al. (2016) [14] to assess an individual’s confidence in discussing sexual topics. Comprising 20 items divided into five subscales—Contraceptive Communication, Negative Sexual Messages, Positive Sexual Messages, Sexual History, and Condom Negotiation—each item is rated on a four-point Likert scale from “very difficult” (1) to “very easy” (4). Higher total scores indicate greater self-efficacy in sexual communication. The scale exhibited strong overall reliability with a Cronbach’s alpha of 0.93. Validity was supported through Exploratory Factor Analysis (EFA), identifying the five-factor structure, and further reinforced by Confirmatory Factor Analysis (CFA). The scale demonstrated high reliability in this study with a Cronbach’s alpha of 0.912. Post-translation into Arabic, validity was confirmed via EFA, showing factor loadings between 0.48 and 0.87, which improved to 0.63 to 0.95 after varimax rotation, exceeding the 0.40 threshold and accounting for 73.814% of the total variance. The Kaiser–Meyer–Olkin (KMO) measure was robust at 0.945, and Bartlett’s test of sphericity was highly significant (*p* ≤ .001), confirming the data’s suitability for factor analysis. Consequently, all items on the scale were retained.

### Emotional intelligence scale

The Trait Meta-Mood Scale (TMMS-24), initially developed by Salovey et al. (1995), is designed to assess trait emotional intelligence through its three dimensions: emotional perception, emotional comprehension, and emotional regulation [24]. Each dimension comprises eight items, rated on a five-point Likert scale from strongly disagree (1) to agree (5) strongly. The total score ranges from 24 to 120, with higher scores indicating more excellent trait emotional intelligence. Espinoza et al. (2015) validated the TMMS-24 among nursing students, demonstrating robust reliability with Cronbach’s alpha values above 0.85 for all dimensions [25]. Confirmatory factor analysis (CFA) validated the scale’s three-factor structure with satisfactory fit indices. In contrast, exploratory factor analysis (EFA) revealed that these factors accounted for 56.5% of the variance, aligning well with the theoretical model. The TMMS-24 showed high reliability in this study with a Cronbach’s alpha of 0.925. After translation into Arabic, EFA confirmed its validity, with factor loadings ranging from 0.50 to 0.90, improving to 0.65 to 0.92 post-varimax rotation and explaining 75.200% of the variance. The Kaiser–Meyer–Olkin (KMO) measure was excellent at 0.940, and Bartlett’s test of sphericity was highly significant (*p* ≤ .001), validating the data’s suitability for factor analysis. All items on the scale were retained.

### Study procedures

#### Tool preparation and pilot study

The research instruments, including SHELA, SCSES, and the Emotional Intelligence Scale, were translated into Arabic by bilingual experts proficient in both English and Arabic, emphasizing accuracy and cultural relevance. To ensure linguistic equivalence, the tools were back-translated into English. Face validity was evaluated by a panel of five experts in community health nursing and psychiatric and mental health nursing, who reviewed each item for clarity, cultural relevance, and conceptual accuracy. The Scale Content Validity Index (S-CVI) for all instruments exceeded 0.90, indicating excellent content validity.

Additionally, a pilot study involving 30 married female nursing students was conducted to assess the clarity and reliability of the Arabic versions. Based on their feedback, no substantial modifications were necessary. Cronbach’s alpha values demonstrated high internal consistency: 0.941 for SHELA, 0.912 for SCSES, and 0.925 for the Emotional Intelligence Scale. Participants in the pilot study were excluded from the main sample to prevent response bias.

### Data collection

Data collection for this study took place between August and September 2024, following the approval of all necessary permissions. A convenience sampling method was used to recruit participants, as the researchers had direct access to the population of interest married female nursing students within the university setting. This non-probability sampling technique was selected based on feasibility and time constraints, with acknowledgment of its limitations regarding generalizability.

Before initiating data collection, the researchers thoroughly explained the study’s objectives to each student, emphasizing the voluntary nature of participation. Written informed consent was obtained from all participants. To promote trust and openness, the researchers assured participants of the confidentiality and anonymity of their responses.

Questionnaires were distributed in quiet and private locations, such as vacant lecture halls and libraries, between 9 a.m. and 2 p.m., from Saturday to Thursday. Participants typically completed the questionnaires within 10 to 15 min.

### Ethical considerations

Approval was obtained from the Research Ethics Committee of the Faculty of Nursing at Tanta University, Egypt, with reference number (2024-9-520 ). The study adhered to ethical principles outlined in the Declaration of Helsinki, protecting participants’ rights and well-being. All participants provided written informed consent following a thorough explanation of the study’s objectives. Participants’ privacy and anonymity were rigorously protected, and all data collected was treated with the utmost confidentiality. Furthermore, participants were informed that they had the right to withdraw from the study at any time.

### Statistical analysis

All statistical analyses were conducted using IBM SPSS Statistics version 28 and the PROCESS macro for mediation analysis. Before analysis, data were inspected for accuracy, as well as the assumptions of normality. Descriptive statistics (means, standard deviations, frequencies, and percentages) were computed to summarize the sample’s demographic characteristics and the key study variables. All tests were two-tailed, and a significance level of *p* < .05 was applied to all statistical analyses unless otherwise specified. Data were presented as means (M) and standard deviations (SD) for continuous variables and as frequencies (%) for categorical variables.

Independent sample t-tests and one-way ANOVA were used to examine differences in Sexual Health Literacy, Sexual Communication Self-Efficacy, and Emotional Intelligence across demographic groups, including age, residence, working status, duration of marriage, number of children, and husband’s characteristics. Pearson correlation coefficients were calculated to examine the relationships between Sexual Health Literacy, Sexual Communication Self-Efficacy, and Emotional Intelligence, as well as their subdomains.

To test the hypothesis that Emotional Intelligence mediates the relationship between Sexual Health Literacy and Sexual Communication Self-Efficacy, mediation analyses were performed using the PROCESS macro (Model 4) developed by Hayes. The indirect effect of Sexual Health Literacy on Sexual Communication Self-Efficacy through Emotional Intelligence was estimated using bias-corrected bootstrapping with 5,000 resamples. The indirect effect was considered significant if the bootstrapped indirect effect’s 95% confidence interval (CI) did not include zero.

## Results

The demographic characteristics of the study sample and their associations with the three studied variables are presented in Table 1. Most studied sample (59.7%) was between 20 and 30 years old. There were no significant differences in Sexual Health Literacy, Sexual Communication Self-Efficacy, or Emotional Intelligence based on age groups. Similarly, participants residing in urban and rural areas did not show significant differences across these three variables. Regarding working status, 94.7% of the participants were working, but there were no significant differences between working and non-working participants regarding the studied variables. When considering the duration of marriage, there were also no significant differences in the studied variables across the three marriage duration categories.

Participants’ age at marriage did not significantly affect Sexual Health Literacy, but Emotional Intelligence was marginally higher among participants who married at a younger age. The number of children did not significantly influence any of the variables. However, participants with more than three children exhibited lower Sexual Health Literacy and Sexual Communication Self-Efficacy, but these differences were not statistically significant. Regarding husband characteristics, participants with husbands aged 20 to 30 years had slightly higher levels of Sexual Health Literacy, but no significant differences were found across the age groups for studied variables. Additionally, participants whose husbands had a university education exhibited slightly higher Sexual Health Literacy (M = 138.31). However, again, no significant differences were observed for the studied variables based on husband education level.

**Table 1:** Sample demographics and differences to studied variables (*N* = 340)

Characteristic		No. (%)	Sexual health literacy		Sexual communication self-efficacy		Emotional intelligence	
			M (SD)	t/F	M (SD)	t/F	M (SD)	t/F
Age	20: <30	203 (59.7)	137.4 (19.8)	F = 0.119	46.79 (9.5)	F = 0.848	84.85 (13.43)	F = 0.752
	30:<40	129 (37.9)	137.13 (19.87)		46.84 (9.4)		86.57 (11.77)	
	*≥* 40	8 (2.4)	140.86 (10.76)		42.14 (4.34)		84.14 (12.64)	
Residence	Urban	120 (35.3)	138.33 (19.26)	t = 0.634	47.45 (10.48)	t = 1.015	84.82 (13.72)	t = 0.673
	Rural	220 (64.7)	136.93 (19.88)		46.37 (8.75)		85.83 (12.27)	
Working status	Not work	18 (15.3)	136.67 (16.91)	t = 0.194	45.94 (7.21)	t = 0.478	89.39 (7.22)	t = 0.634
	Work	322 (94.7)	137.47 (19.81)		46.8 (9.51)		85.25 (13)	
Duration of marriage	< 5	116 (34.1)	138.11 (19.42)	F = 0.739	47.57 (9.29)	F = 0.163	85.96 (12.51)	F = 1.337
	*≥* 5 : <10	146 (42.9)	138.15 (21)		47.02 (9.34)		84.92 (13.22)	
	*≥* 10	78 (22.9)	135.05 (17.26)		45.03 (9.56)		85.78 (12.49)	
Age of marriage	< 20	21 (6.2)	140.1 (15.1)	F = 0.345	46.86 (9.03)	F = 2.155	91.33 (8.78)	F = 2.551
	20:<30	251 (73.8)	137.58 (19.47)		45.75 (9.23)		85.31 (12.64)	
	30:<40	7 (2.1)	142.14 (17.62)		52.86 (5.81)		89 (7.83)	
Family number	0	15 (4.4)	137.2 (15.6)	F = 0.496	48.8 (9.68)	F = 1.703	87.07 (9.38)	F = 0.825
	1	77 (22.6)	139.14 (16.73)		47.26 (9.33)		85.96 (12.08)	
	2	146 (42.9)	137.97 (21.31)		46.62 (9.86)		84.68 (14.12)	
	3	73 (21.5)	136.48 (16.92)		46.67 (8.19)		87.75 (10.17)	
	> 3	29 (8.6)	131.77 (25.03)		40.46 (9.39)		83.85 (16.49)	
Husband age	20: <30	70 (20.6)	140.54 (17.42)	F = 1.166	47.66 (8.91)	F = 0.656	86.84 (12.58)	F = 0.490
	30: <40	214 (62.9)	136.63 (20.5)		46.21 (9.71)		85.26 (12.79)	
	40 and more	53 (17.5)	139.05 (17.37)		46.98 (9.04)		86.5 (12.49)	
Husband’ education	Basic	19 (5.6)	133 (18.78)	F = 0.940	47.11 (12.11)	F = 0.070	84.68 (10.96)	F = 0.242
	Secondary education	88 (25.9)	136.03 (24.18)		46.44 (9.15)		86.26 (14.14)	
	University education	233 (68.5)	138.31 (17.73)		46.84 (9.28)		85.24 (12.42)	

t = Independent sample t test, F = ANOVA test, M = Mean, SD = Standard deviation, P = Significance at < 0.05

Table 2 presents that the three subdomains of Sexual Health Literacy—Reading and Understanding, Evaluation and Application of Information, and Skills of Access—were all highly and positively correlated with each other and with the Total Sexual Health Literacy score. Specifically, Reading and Understanding showed strong positive correlations with Evaluation and Application of Information (*r* = .717, *p* < .01) and Skills of Access (*r* = .620, *p* < .01).

Total Sexual Health Literacy was also positively correlated with all subdomains of Sexual Communication Self-Efficacy. The strongest correlations were observed between Total Sexual Health Literacy and Contraceptive Communication (*r* = .339, *p* < .01) and Total Sexual Communication Self-Efficacy (*r* = .388, *p* < .01). The subdomains of Sexual Communication Self-Efficacy were significantly interrelated. Contraceptive Communication was strongly correlated with Negative Sexual Messages (*r* = .612, *p* < .01), Positive Sexual Messages (*r* = .366, *p* < .01), Sexual History (*r* = .440, *p* < .01), and Condom Negotiation (*r* = .417, *p* < .01). Additionally, Total Sexual Communication Self-Efficacy was highly correlated with its subdomains, with correlations ranging from *r* = .725 (Contraceptive Communication) to *r* = .839 (Sexual History), all significant at *p* < .01.

Emotional Intelligence and its subdomains also showed significant positive correlations with both Sexual Health Literacy and Sexual Communication Self-Efficacy. Total Emotional Intelligence was moderately correlated with Total Sexual Health Literacy (*r* = .560, *p* < .01) and Total Sexual Communication Self-Efficacy (*r* = .319, *p* < .01). Among the subdomains, Emotional Perception had the strongest correlation with Total Sexual Health Literacy (*r* = .488, *p* < .01). Furthermore, Emotional Regulation and Emotional Comprehension were positively correlated with various subdomains of Sexual Communication Self-Efficacy. Emotional Regulation correlated significantly with Contraceptive Communication (*r* = .215, *p* < .01) and Negative Sexual Messages (*r* = .243, *p* < .01), while Emotional Comprehension showed significant correlations with Positive Sexual Messages (*r* = .181, *p* < .01) and Condom Negotiation (*r* = .147, *p* < .01). Overall, the correlation analysis indicated that Sexual Health Literacy, Sexual Communication Self-Efficacy, and Emotional Intelligence were interrelated constructs.

**Table 2:** Correlation matrix of studied variables and their subdomains (*n* = 340)

Variables	1	2	3	4	5	6	7	8	9	10	11	12	13	14
Reading and understanding	1													
Evaluation and application of information	0.717**	1												
Skills of access	0.620**	0.754**	1											
Total sexual health literacy	0.910**	0.906**	0.854**	1										
Contraceptive communication	0.335**	0.237**	0.323**	0.339**	1									
Negative sexual messages	0.298**	0.239**	0.287**	0.311**	0.612**	1								
Positive sexual messages	0.293**	0.266**	0.295**	0.320**	0.366**	0.540**	1							
Sexual history	0.280**	0.176**	0.272**	0.277**	0.440**	0.569**	0.556**	1						
Condom negotiation	0.314**	0.167**	0.257**	0.286**	0.417**	0.470**	0.575**	0.751**	1					
Total sexual communication Self-efficacy	0.386**	0.274**	0.364**	0.388**	0.725**	0.808**	0.763**	0.839**	0.814**	1				
Emotional perception	0.406**	0.440**	0.478**	0.488**	0.197**	0.280**	0.282**	0.263**	0.245**	0.320**	1			
Emotional regulation	0.446**	0.434**	0.440**	0.493**	0.215**	0.243**	0.261**	0.225**	0.235**	0.298**	0.681**	1		
Emotional Comprehension	0.453**	0.407**	0.431**	0.485**	0.160**	0.227**	0.181**	0.131*	0.147**	0.214**	0.578**	0.659**	1	
Total Emotional intelligence	0.499**	0.490**	0.516**	0.560**	0.219**	0.287**	0.278**	0.238**	0.241**	0.319**	0.866**	0.899**	0.850**	1

**Fig. 1:**
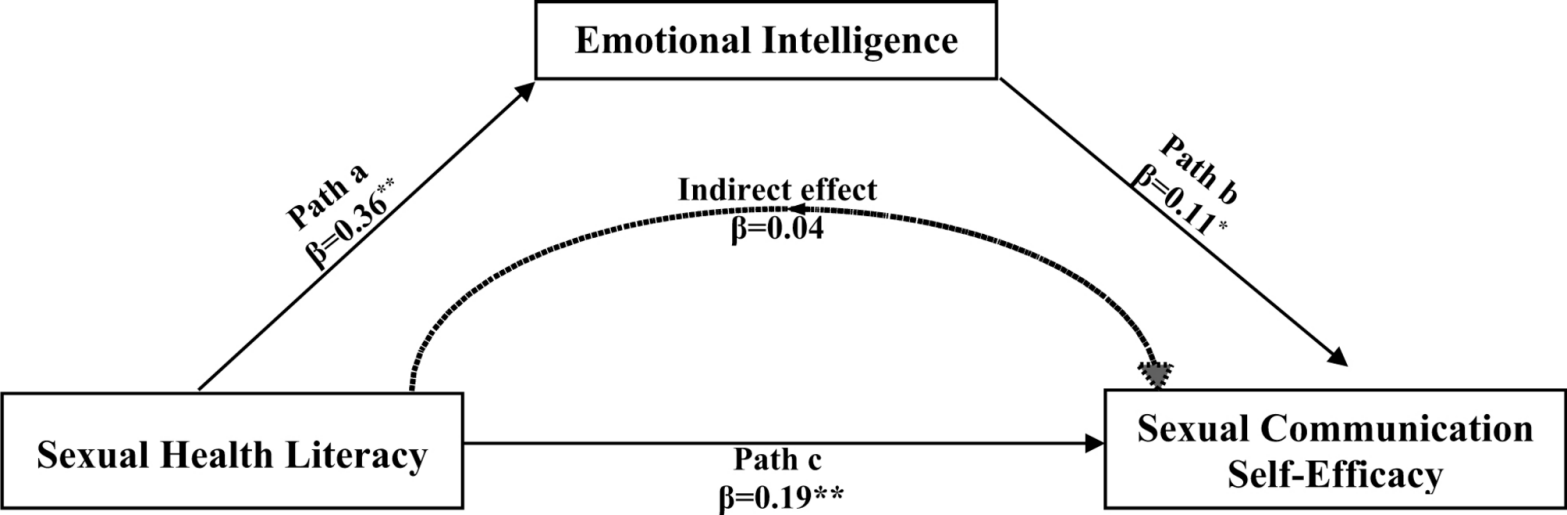
Mediation analysis showing Emotional Intelligence as a mediator between Sexual Health Literacy and Sexual Communication Self-Efficacy ( *n* = 340)

Table 3; Fig. 1 presents the results of the mediation analysis that revealed that Sexual Health Literacy had a significant positive effect on Emotional Intelligence (B = 0.36, SE = 0.03, t = 12.44, *p* < .001), indicating that higher levels of Sexual Health Literacy were associated with increased Emotional Intelligence among the participants. Regarding the direct effects, Sexual Health Literacy also had a significant positive relationship with Sexual Communication Self-Efficacy (B = 0.15, SE = 0.03, t = 5.08, *p* < .001), even after accounting for the influence of Emotional Intelligence. This indicates that participants with higher levels of Sexual Health Literacy were more likely to demonstrate greater confidence and capability in communicating about sexual health, independent of their emotional intelligence. Furthermore, Emotional Intelligence was found to significantly influence Sexual Communication Self-Efficacy (B = 0.11, SE = 0.04, t = 2.47, *p* = .014), demonstrating that higher levels of emotional intelligence contributed to improved communication efficacy in sexual matters.

The total effect of Sexual Health Literacy on Sexual Communication Self-Efficacy was also significant (B = 0.19, SE = 0.02, t = 7.75, *p* < .001), confirming that Sexual Health Literacy played a substantial role in determining communication self-efficacy. The indirect effect of Sexual Health Literacy on Sexual Communication Self-Efficacy, mediated through Emotional Intelligence, was significant (B = 0.04), indicating that Emotional Intelligence partially mediated the relationship between Sexual Health Literacy and Sexual Communication Self-Efficacy, suggesting that the effect of Sexual Health Literacy on communication self-efficacy was, in part, explained by its ability to enhance Emotional Intelligence. Overall, these findings suggest that Sexual Health Literacy exerts both direct and indirect effects on Sexual Communication Self-Efficacy, with Emotional Intelligence serving as an important mediator. The model accounted for a substantial portion of the variance in Emotional Intelligence (R² = 0.31, F = 154.76, *p* < .001) and Sexual Communication Self-Efficacy (R² = 0.17, F = 33.54, *p* < .001), providing a robust explanation of the relationships between these variables.

**Table 3:** Mediation analysis of emotional intelligence as a mediator in the relationship between sexual health literacy and sexual communication Self-Efficacy (*n* = 340)

Effect	B	SE	t	*p*	LLCI	ULI	Standardized B
Outcome: Emotional Intelligence (Mediator)^*^							
Constant	35.35	4.07	8.68	0.000	27.34	43.35	-
Sexual Health Literacy	0.36	0.03	12.44	0.000	0.31	0.42	0.56
Outcome: Sexual Communication Self-Efficacy (Direct & Indirect Effects)^**^							
Constant	17.37	3.65	4.75	0.000	10.18	24.55	-
Sexual Health Literacy (Direct)	0.15	0.03	5.08	0.000	0.09	0.20	0.31
Emotional Intelligence (Mediator)	0.11	0.04	2.47	0.014	0.02	0.20	0.15
Total Effect of Sexual Health Literacy on Sexual Communication Self-Efficacy^***^							
Sexual Health Literacy (Total)	0.19	0.02	7.75	0.000	0.14	0.23	0.39
Indirect Effect through Emotional Intelligence							
Sexual Health Literacy → Emotional Intelligence → Sexual Communication Self-Efficacy	0.04	0.02	-	-	0.00	0.07	0.08

## Discussion

This study aimed to examine the relationships between sexual health literacy, emotional intelligence, and sexual communication self-efficacy among married nursing students and assess the mediating role of emotional intelligence. Initially, the findings revealed that sexual health literacy significantly influences sexual communication self-efficacy among married nursing students. This critical finding suggests that when nursing students possess greater knowledge and understanding of sexual health, they are more likely to feel confident and capable in discussing sexual health matters with their partners. The findings emphasize the importance of considering both personal and professional contexts in designing effective sexual health programs. Increased sexual health literacy provides individuals with accurate information, reduces misconceptions, and alleviates anxiety around discussing sensitive topics, thereby boosting their confidence in initiating and maintaining open dialogues about sexual health [25]. This finding aligns with Fuzzell et al. (2017) [26] and Jayasundara (2020) [27] and in contrast to Edison et al. (2021) [28].

Furthermore, the study findings revealed that sexual health literacy has the potential to enhance married nursing students’ emotional intelligence. This may be due to the fact that increased sexual health literacy not only equips students with essential knowledge but also promotes greater self-awareness and emotional regulation [29]. This finding advances the literature by uncovering the unique dynamics of sexual health literacy and emotional intelligence in nursing students, offering a more comprehensive understanding of these constructs, and highlighting practical applications for both professional and personal development. These contributions fill critical gaps in the existing knowledge and provide a foundation for future research and interventions in this area. This finding is in accordance with Milani et al. (2019) [30] and Ball et al. (2022) [31].

The study findings also showed that emotional intelligence of married nursing students increase their sexual communication self-efficacy. This is an interesting result add to the literature by highlighting the benefits of fostering emotional intelligence among married nursing students. Students with higher emotional intelligence are better equipped to manage their emotions, express themselves clearly [32], and navigate potentially sensitive conversations with their partners [33]. Additionally, emotional intelligence helps individuals recognize and regulate both their own emotions and those of others, promoting effective communication and reducing anxiety in intimate discussions [34]. As a result, this strengthens their sexual communication self-efficacy. In comparison with previous studies, our results match with Morales Rodríguez et al. (2020) [35] and contrast with Willi and Burri (2018) [36].

Lastly, the findings of this study suggest that emotional intelligence serves as a mediator in the relationship between sexual health literacy and sexual communication self-efficacy among married nursing students. This implies that when students enhance their sexual health literacy, it not only improves their knowledge but also fosters the development of emotional intelligence, which in turn boosts their confidence and ability to engage in effective sexual communication. These results corroborate previous studies, which showed that enhancing sexual health knowledge improves individuals’ emotional skills [5, 37], enabling more confident and effective communication in sexual contexts [38].

This study adds to the literature by its focus on married nursing students, a population that has been largely overlooked in prior research on sexual health literacy and emotional intelligence. By targeting this unique group, the study uncovers how their dual roles—professional training in healthcare and personal responsibilities in marriage—influence sexual health outcomes in ways not previously explored. Specifically, our findings demonstrate that emotional intelligence plays a critical mediating role in this context, suggesting that the interplay of professional knowledge and personal life amplifies its impact on sexual communication self-efficacy. This contributes to the existing literature by emphasizing the need for context-specific research in sexual health studies. Furthermore, these insights add to the field by offering practical implications: educational programs tailored for healthcare trainees, particularly those balancing personal and professional demands, could integrate emotional intelligence training alongside sexual health literacy to enhance communication skills. This approach could improve both their personal well-being and their future effectiveness as healthcare providers.

## Conclusion

The study demonstrated that Sexual Health Literacy significantly positively affects both Emotional Intelligence and Sexual Communication Self-Efficacy among married nursing students. Participants with higher Sexual Health Literacy were more emotionally intelligent and demonstrated greater confidence in communicating about sexual health. Emotional Intelligence partially mediated the relationship between Sexual Health Literacy and Sexual Communication Self-Efficacy, highlighting its role in improving communication efficacy. These findings emphasize the value of enhancing Sexual Health Literacy to boost Sexual Communication Self-Efficacy directly and through Emotional Intelligence. This suggests that educational interventions focusing on improving Sexual Health Literacy and Emotional Intelligence could effectively empower married nursing students to engage in more confident and effective sexual communication.

### Implications

The findings of this study have important implications for nursing research, education, and practice. Our results demonstrated that Emotional Intelligence significantly mediates the relationship between Sexual Health Literacy and Sexual Communication Self-Efficacy, highlighting the crucial role of emotional regulation in effective sexual health discussions. In terms of research, future studies should build on these findings by developing and evaluating interventions that simultaneously enhance both Sexual Health Literacy and Emotional Intelligence to improve Sexual Communication Self-Efficacy, particularly among married women. Further investigations across diverse populations and healthcare settings will help determine the generalizability of these results and identify additional psychosocial factors that may influence sexual health communication.

In nursing education, our findings suggest that strengthening Emotional Intelligence could enhance nursing students’ ability to effectively communicate about sexual health topics. Therefore, integrating targeted training on Sexual Health Literacy and Emotional Intelligence into nursing curricula could better prepare students to confidently address sexual health concerns in both personal and professional contexts. Nurse educators should incorporate structured modules on emotional regulation, sexual communication strategies, and patient-centered sexual health discussions to improve students’ competency in this area.

From a practical standpoint, our study underscores the need for nurses to recognize the interplay between Emotional Intelligence and Sexual Health Literacy when addressing patients’ sexual health concerns. Healthcare providers should be trained to assess and support both the cognitive (literacy) and emotional (self-efficacy) aspects of sexual health discussions. By fostering a supportive environment that enhances patients’ confidence in discussing sexual health, nurses can play a pivotal role in improving overall sexual well-being.

### Strengths and limitations

This study’s strengths lie in its rigorous methodological design, beginning with a well-validated cross-sectional survey approach following the STROBE checklist. The use of standardized and reliable measurement tools, such as the Sexual Health Literacy for Adults (SHELA), the Sexual Communication Self-Efficacy Scale (SCSES), and the Trait Meta-Mood Scale (TMMS-24) for emotional intelligence, ensures robust data collection. The psychometric properties of these instruments, including high Cronbach’s alpha values and confirmed validity through factor analysis, enhance the reliability of the findings. Additionally, the study’s large sample size of 340 participants, determined using power analysis, improves the generalizability of the results to the population of married nursing students. The pilot study further adds to the study’s rigor by validating the instruments in the Arabic context before the main study.

However, the study has some limitations. First, the cross-sectional design precludes the establishment of causal relationships, limiting the interpretation of how variables like emotional intelligence and sexual communication self-efficacy influence sexual health literacy. Longitudinal studies would be better suited to explore these relationships over time. Additionally, the exclusion of participants with severe mental health disorders may reduce the generalizability of the findings to a broader population of nursing students, as emotional intelligence and communication self-efficacy could be particularly important in students with such conditions. Finally, self-reported measures, while helpful, carry the risk of social desirability bias, potentially influencing participants’ responses and overestimating their sexual health literacy or communication skills.

## Data Availability

The datasets generated and analyzed during the current study are not publicly available due to confidentiality agreements but are available upon reasonable request from the corresponding author.
